# A Practice Algorithm for Distinguishing Uterine Arteriovenous Malformation in Postpregnancy Hemorrhage

**DOI:** 10.1155/carm/2450436

**Published:** 2025-12-22

**Authors:** Sadhvi Batra, Mario Dervishi, Mohamed Al-Natour, Tyler Katz

**Affiliations:** ^1^ Division of Gynecologic Oncology, University of Michigan, Ann Arbor, 48109, Michigan, USA, umich.edu; ^2^ Division of Interventional Radiology, UH Cleveland Medical Center, Cleveland, 44106, Ohio, USA, uhhospitals.org; ^3^ Department of Obstetrics and Gynecology, UH Cleveland Medical Center, Cleveland, 44106, Ohio, USA, uhhospitals.org

## Abstract

**Introduction:**

Diagnosing the cause of delayed postpregnancy hemorrhage is imperative for effective management. The most common factor to lead to this is retained products of conception (RPOC); however, it is often difficult to distinguish RPOC from uterine arteriovenous malformations (AVMs). AVMs are abnormal vascular connections that can lead to sudden and persistent uterine bleeding that can be life‐threatening. Though rare, there are many challenges to establishing a true diagnosis of an AVM versus RPOC and planning for treatment when a diagnosis of AVM is suspected.

**Methods:**

In this study, authors present a case series of five patients who had the potential diagnosis of an AVM and how each was managed. The authors extrapolated best practices from these five cases and created a new algorithm to tailor management for patients with concern for AVMs.

**Results:**

This algorithm relies on utilizing transvaginal ultrasound as the first diagnostic technique, facilitating an early conversation with radiology if suspicion for AVM exists, and then determining if further imaging is needed or if the clinician can proceed to diagnostic hysteroscopy. In addition, if the decision is made for hysteroscopy, consultation with interventional radiology is recommended in case of urgent intervention to prevent further morbidity with hysterectomy.

**Conclusion:**

Though much is left to be explored regarding conservative management for uterine AVM, this paper is an initial attempt at proposing a practice algorithm for multidisciplinary management of this potentially life‐threatening condition.

## 1. Introduction

The most common cause of delayed postpregnancy hemorrhage is retained products of conception (RPOCs) [1]. However, more recently imaging studies are finding challenging to delineate between other causes of postpregnancy hemorrhage that have similar characteristics on ultrasound such as uterine artery pseudoaneurysm (UAP)—one layered vascular abnormalities with turbulent blood flow—and uterine arteriovenous malformations (AVMs)—abnormal connections between arteries and veins within the uterine tissue [1, 2]. These vascular malformations can lead to persistent, abnormal bleeding and pose a risk for serious complications, including hemorrhage. Nonetheless, distinguishing between the two diagnoses accurately can help guide effective management and prevent further morbidity.

Diagnosis for any of these conditions is suspected based on imaging modalities such as transvaginal ultrasound (TVUS), computed tomography (CT) angiogram, magnetic resonance imaging (MRI), or conventional angiography [1, 3]. Management of either of these conditions requires accurate diagnosis. RPOCs require removal of tissues, whereas in the case of AVM, instrumentation risks worsening the hemorrhage.

In this article, we present and discuss five different cases. Patients 1 and 2 were diagnosed with AVM based on imaging and underwent uterine artery embolization (UAE). Patient 3 was diagnosed with RPOC and underwent prophylactic UAE. Patients 4 and 5 were diagnosed with RPOC and underwent medical treatment and hysteroscopy respectively. See Supporting Information (available here) for table representation of cases.

The aim of this paper is to distinguish between an AVM versus RPOC based on clinical suspicion and imaging features and propose a treatment algorithm for both obstetricians and gynecologists (OBGYNs) and interventional radiologists (IRs) to better guide clinical treatment. Institutional Review Board approval was obtained on 11/13/2023, STUDY20231300.

## 2. Case Series

### 2.1. Case 1

**Figure 1:**
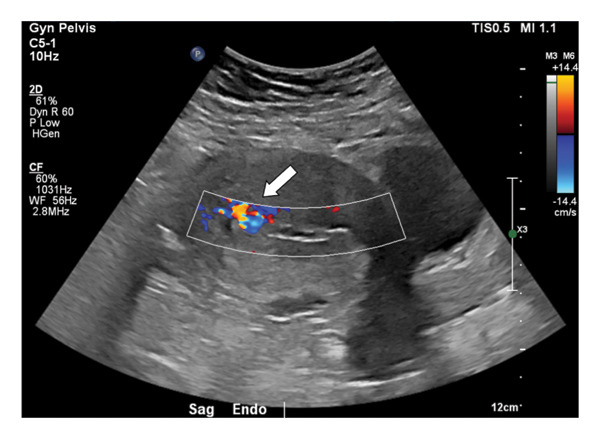
Transvaginal ultrasound demonstrating intrauterine color Doppler flow concerning for hypervascular RPOC or AVM.

**Figure 2:**
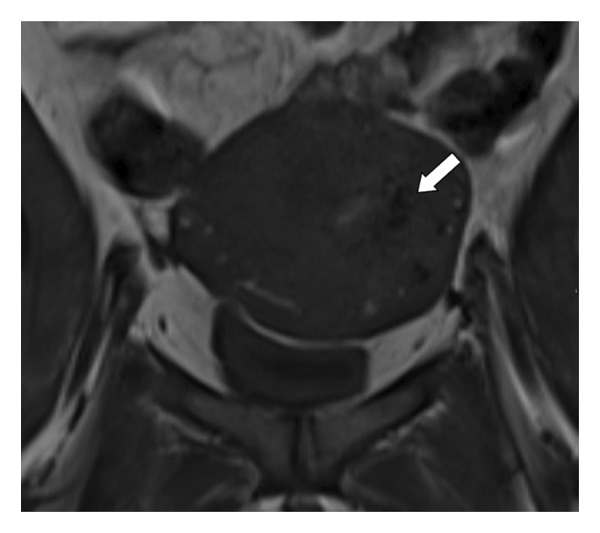
Coronal MRI of the pelvis (T1‐weighted) shows multiple serpentine flow signal voids in the left uterine wall, endometrial cavity, and parametrium features concerning for uterine arteriovenous malformation.

**Figure 3:**
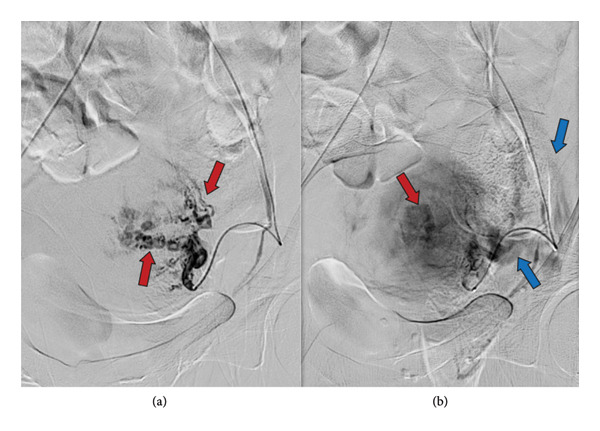
Left uterine artery angiogram (a, b), showing small serpentine branches (red arrow) with early venous return (blue arrows) confirming the diagnosis of AVM.

A 41‐year‐old G3P1021 with a history of prior cesarean delivery and dilation and curettage (D&C) for a 10‐week missed abortion, presented to our institution after 4 weeks with intractable bleeding following the D&C. RPOC or uterine AVM was suspected, and a TVUS was performed suspicious for uterine AVM, see Figure 1. To delineate between RPOCs or AVM, an MRI of the pelvis was performed demonstrating features concerning for uterine AVM, see Figure 2. Due to ongoing bleeding and hemoglobin dropping from baseline of 12 to 9 g/dL, Interventional Radiology was consulted for diagnostic angiogram with possible angioembolization. Angiography confirmed a uterine AVM, see Figure 3. Particle embolic spheres were used with successful embolization. The patient did well after the procedure with resolution of her bleeding and was discharged home the next day with short‐term clinic follow‐up.

### 2.2. Case 2

**Figure 4:**
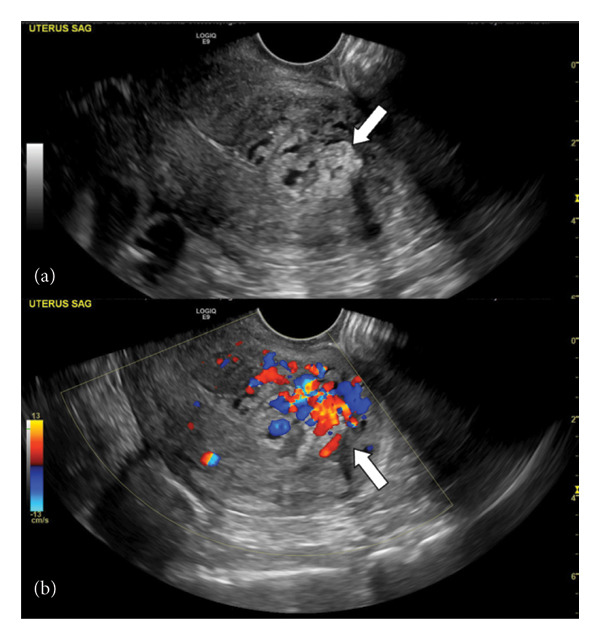
Abdominal ultrasound of the uterus (sagittal view): gray‐scale (a) demonstrates mixed echogenicity content (arrow) with color Doppler flow (b), concerning for uterine AVM.

**Figure 5:**
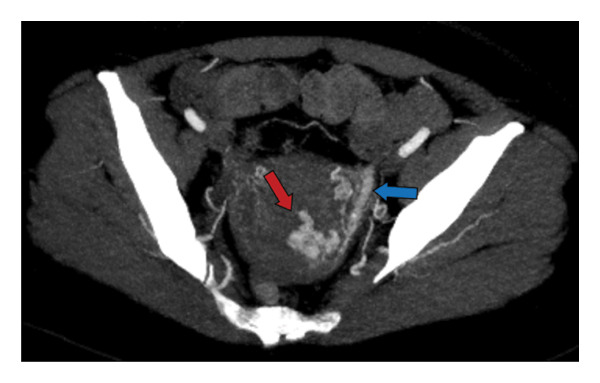
Maximum intensity projection (MIP) axial‐view computed tomography (CT) angiogram of the pelvis demonstrating multiple enhancing left uterine artery branches (red arrow) with early venous drainage (blue arrow), consistent with AVM.

**Figure 6:**
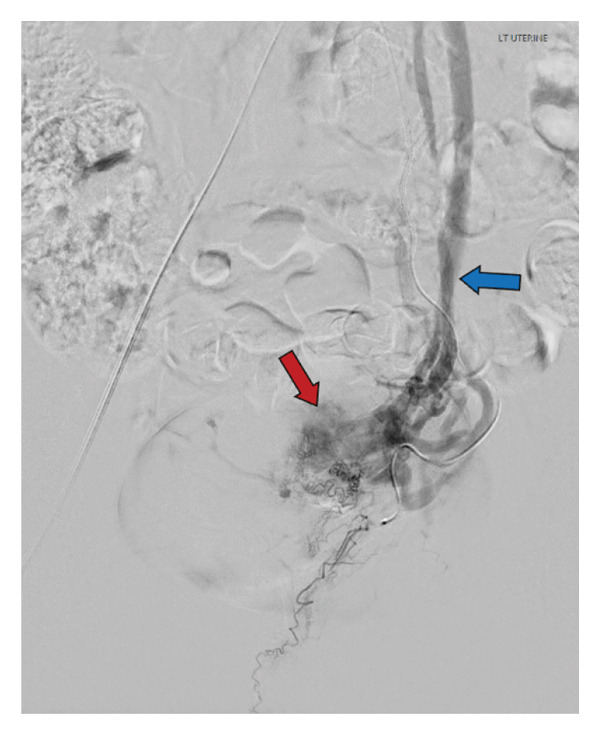
Left uterine artery angiogram confirming CTA findings, showing multiple small serpentine branches (red arrow) arising from the uterine artery with early venous return (blue arrow).

A 35‐year‐old G2P2002 with a history of full‐term, spontaneous vaginal delivery complicated by manual placental removal and postpartum hemorrhage, discharged postpartum Day 2, presented on postpartum Day 4 with light vaginal bleeding and lightheadedness. Vitals were notable for tachycardia. Lab findings showed a hemoglobin level of 10.5 g/dL. Her symptoms resolved with fluid resuscitation, and she was discharged home. On postpartum Day 10, the patient presented again with vaginal bleeding. Further workup with ultrasound imaging was concerning for RPOC, and the patient underwent D&C complicated by post procedural hemorrhage of 1.2 L requiring 4 units of packed RBC and 3 units of fresh frozen plasma with eventual clinical stability and discharge on postoperative Day 4. Pathology from the procedure demonstrated products of conception with Grade 1 focal placental accreta. The patient presented again on postpartum Day 27 with sudden gush of blood. CT angiogram and TVUS were consistent with AVM (Figures 4 and 5), and interventional radiology was consulted for UAE. Angiogram confirmed left‐sided AVM with successful embolization, see Figure 6. The patient’s bleeding resolved to light spotting. She was discharged home the next day with close follow‐up scheduled.

### 2.3. Case 3

A 32‐year‐old G2P2 with a history of vaginal delivery complicated by cervical laceration who underwent exam under anesthesia for repair. During examination, retained placental tissue was noted to be adherent to the myometrium in the anterior lower uterine segment, concerning for placenta accreta spectrum. A biopsy of the tissue was sent for pathology review and confirmed to be RPOC. She was initially managed conservatively due to minimal bleeding and discharged home routinely with ample precautions and close follow‐up. On postpartum Day 16, the patient reported continued bleeding, and TVUS was performed showing concern for RPOCs. Due to the prior intraoperative findings, decision was made to refer to IR. PThe patient subsequently underwent angioembolization with IR to avoid hemorrhage. Postembolization bleeding completely resolved.

### 2.4. Case 4

A 23‐year‐old G3P1021 with a history of spontaneous abortion at 6‐week gestation who presented for continued vaginal bleeding for one month since abortion. On arrival, the patient was hemodynamically stable with a hemoglobin level of 11.1 g/dL and beta‐human chorionic gonadotropic hormone (Beta‐HCG) of 165 mLU/mL. TVUS showed a 8.2‐cm uterus with thickened endometrium and focal heterogenous area of turbulent color flow at the junction of the endometrium and myometrium, concerning for RPOC or possibly AVM.

Clinical suspicion was higher for RPOC; therefore, she was discharged home with misoprostol. On one‐week follow‐up in clinic, the patient had experienced only scant bleeding, and the ultrasound showed a thin endometrial suggesting passage of products of conception.

### 2.5. Case 5

A 34‐year old G1P1 with a history of full‐term vaginal delivery complicated by retained placenta requiring manual removal of placenta and D&C who presented postpartum Day 24 for heavy vaginal bleeding.

The patient was hemodynamically stable with normal hemoglobin. Ultrasound was performed showing thickened endometrium with hypervascular, heterogenous products in the endometrial canal concerning for RPOC.

Images were reviewed with IR who stated that there was low suspicion for AVM.

The patient subsequently underwent hysteroscopy with direct visualization and resection of RPOC and eventual resolution of bleeding.

## 3. Discussion

### 3.1. Establishing a Practice Algorithm

Patients who present with delayed postpregnancy hemorrhage can be a clinical conundrum.

**Figure 7:**
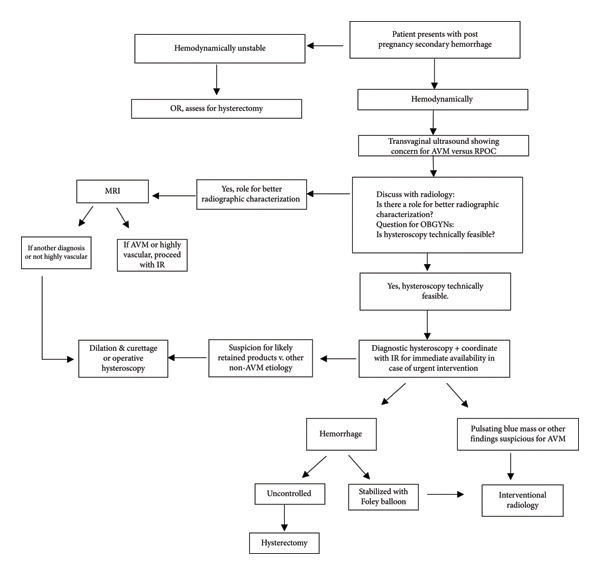
The algorithm for approaching postpregnancy secondary hemorrhage.

Often, imaging to assess the etiology of bleeding is indeterminate, and it includes a differential diagnosis of uterine AVM or RPOCs for which the treatment differs. Using the case series above, the authors extrapolated a practice algorithm below to guide multidisciplinary management, see Figure 7.

AVMs that are acquired often occur after some form of instrumentation of the uterus such as a D&C for postpartum hemorrhage or surgical abortion [1]. The cases mentioned above suggest that diagnostic radiology attempts to delineate them almost routinely on TVUS in order to guide clinicians toward best management practices. However, given the rarity of AVMs and the potentially catastrophic consequences of erroneous diagnosis, the question remains on how to best characterize a true AVM versus an alternative diagnosis to guide the most effective, least invasive treatment for patients.

The algorithmic approach begins with a patient who presents with postpregnancy secondary hemorrhage, defined as vaginal bleeding occurring 24 h after delivery or abortion within a 6‐week period. Patients should initially be assessed for hemodynamic status. If deemed unstable, the authors suggest going straight to the operating room for surgical management with a possibility of hysterectomy.

If the patient is hemodynamically stable, the authors would suggest next obtaining a TVUS as first line for imaging. If this shows concern for AVM versus RPOC, OBGYNs should consider discussing the case with radiology to determine if further imaging can help delineate the diagnosis, or if hysteroscopy is technically feasible with operating room availability, patient consent, and physician comfort.

If radiology suggests that better characterization of the lesion is needed, the imaging most likely to be recommended is MRI. If the MRI shows an AVM or if there is concern that the lesion is highly vascular, the authors recommended proceeding to consult IR to assess for embolization. Nonetheless, if another diagnosis is evident from MRI or if the lesion is not highly vascular based on imaging, the authors suggest proceeding with diagnostic hysteroscopy and possibly D&C.

If OBGYNs deem that hysteroscopy is technically feasible, the authors recommend proceeding with diagnostic hysteroscopy while simultaneously coordinating the procedure with IR availability such that if urgent intervention was needed, surgeons and IR can still provide a conservative treatment option to the patient.

The algorithm then proceeds to delineate management based on hysteroscopic findings. If the surgeons find that suspicion is likely for RPOCs or other etiology, then it is safe to proceed with D&C. However, if hemorrhage is encountered during hysteroscopy, attempts should be made to tamponade the bleeding with a Foley balloon. If unable or this fails, then the authors recommend proceeding with hysterectomy. If able to tamponade with the Foley balloon or in the case of strong suspicion of AVM upon trial of hysteroscopy, the authors recommend involving IR for evaluation for embolization.

**Figure 8:**
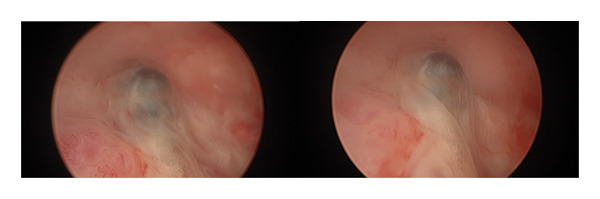
Hysteroscopic appearance of AVM showing pulsating blue mass.

Of note, there are only case reports describing the appearance of an AVM on hysteroscopy at this time. The authors Gingold and Bradley described a “pulsating, blue mass” as the appearance of an AVM on hysteroscopy [3], see Figure 8 for hysteroscopic appearance of AVM.

### 3.2. Imaging Role

Imaging is essential in diagnosing uterine AVMs. Grayscale ultrasound findings usually reveal an irregular mass consisting of cystic and tubular structures in the myometrium and endometrium. Color Doppler examination often shows high‐speed flow in multiple directions and displays color mosaic patterns with low resistance indices [3, 4].

CT angiography and pelvic angiography reveal enlarged uterine arteries, multiple smaller branches with a twisted mass or hypervascularity lesion appearance of the uterine tissue, with early drainage into enlarged veins [3, 4].

MRI and three‐dimensional CT angiography are also valuable diagnostic tools. MRI typically depicts multiple serpentine flow‐related signal voids seen in the uterine wall, endometrial cavity, and parametrium on T1‐ and T2‐weighted images. Contrast‐enhanced dynamic MR angiography can depict complex serpentine abnormal vessels that enhance as intensely as normal vessels and show early venous return [5].

A diagnostic pitfall includes highly vascular RPOC, which can be mistaken for a true AVM of the uterus [4, 6]. Such nonspecific imaging features contribute to the overdiagnosis of uterine AVMs [6].

RPOC demonstrates mixed echogenicity heterogeneous material on grayscale with avascularity, minimal vascularity, or hypervascularity on color Doppler [6]. In instances of highly vascular RPOCs, which can be mistaken for a true AVM of the uterus, MR angiogram or CT angiogram should be performed to differentiate between the two. Accurate identification of AVM on imaging is crucial since endometrial curettage can significantly worsen bleeding in this vascular anomaly [6].

### 3.3. Role for Beta‐HCG

For the diagnosis of AVM, the use of β‐HCG is not used. In one prospective study evaluating the role of β‐HCG as an adjunct to imaging for the diagnosis of RPOC, serum β‐HCG was found not to have a role in the diagnosis of RPOC, as most of the women who had pathologic findings of retained products did not have a detectable HCG time of intervention [7]. As such, this lab value cannot be used to delineate between RPOC and AVM.

### 3.4. Role for D&C Hysteroscopy

The role for D&C along with diagnostic hysteroscopy remains limited when the diagnosis of AVM is highly suspected. Though a diagnostic hysteroscopy can be used to visualize an AVM and differentiate it from another condition presenting similarly, such as RPOC, the challenge with D&C is the risk of disrupting a possible AVM and causing a massive hemorrhage [8].

During hysteroscopy, AVMs have been described to appear as a pulsating blue mass. However, more diagnostic studies visualizing AVMs directly are needed, along with surgeon experience, to identify malformations accurately before they can be definitively characterized, as only one case report to date has described the appearance of AVMs via hysteroscopy [9].

### 3.5. Treatment

#### 3.5.1. Observation

There have been cases described of spontaneous regression of AVMs, though exact characterization of which AVMs may resolve has not been fully described [10].

#### 3.5.2. Medical Management

Given that AVMs most often present with heavy vaginal bleeding, generally conservative management with therapies such as progesterone has not been well established as acute intervention is often needed.

One prospective observational study conducted in postabortal patients with AVMs showed that almost 57% of patients had complete resolution of bleeding after a single 3‐week course of progesterone therapy with norethistrone, and an additional 43% needed another course of treatment prior to complete resolution. However, this study was small and included only 30 patients. Furthermore, the authors of this study noted that patients that responded to medical therapy had a peak systolic velocity (PSV) of the AVM on average around 67.4 cm/s, suggesting a role for measuring PSV in the treatment of AVMs [10].

#### 3.5.3. IR‐Guided Embolization

In patients with massive bleeding or a high risk of bleeding, UAE is the least invasive, effective option with hysterectomy reserved for patients with massive hemorrhage who are hemodynamically unstable for angiointervention [3].

UAE is performed in the angiography suite through femoral or radial artery access under fluoroscopic guidance. A catheter is used to select each uterine artery and bilateral embolization is performed. The type of embolic agent used depends on the patient presentation and etiology. In cases such as postpartum hemorrhage, temporary embolic agent such as gelatin‐sponge particles are most commonly used. Embolic microspheres can be used in combination with the gelfoam or as a single‐treatment agent. In the setting of uterine fibroids embolization (UFE), typically embolic microspheres of various sizes (300–500 microns) are used. In treatment of AVMs when a draining vein is obvious, coils are used to avoid distal migration of embolic agents. Although microsphere can be used in cases that risk of nontargeted embolization is insignificant, technical success is usually defined as nonopacification of the uterine AVMs at the completion pelvic arteriogram with clinical success evidently defined as the cessation of abnormal uterine bleeding or minimal estimated blood loss on subsequent clinical follow‐up with no AVM by ultrasound examination [11].

A point of discussion between interventionalists, OBGYNs, and patients has been the long‐term effects of UAE on the rate of future fertility. To this date, there are no randomized controlled trials on the material used during embolization or the effects of embolization alone on future fertility. Associations between pregnancy rates and some potentially relevant factors, such as bilateral versus unilateral embolization, number of embolization sessions, and type of embolizing agents and size, remain controversial because of lack of data [11, 12]. Furthermore, nonmodifiable patient factors such as age of patients and prior medical history (i.e., spontaneous abortions) are also an important part of the discussion between providers and patients when UAE is offered as a treatment option.

Nevertheless, based on the available data, a recently published systematic review and meta‐analysis by Ghanati et al. on “Pregnancy Rate and Outcomes following Uterine Artery Embolization for Uterine Arteriovenous Malformations” fertility does not appear to be substantially compromised by UAE for uterine AVM, and the pooled estimate of live birth rate appears similar to that of the general population [12].

Additionally, multiple studies regarding uterine artery embolization that have been conducted in the setting of uterine fibroids, which have shown that following UFE, pregnancy is attainable, and many of these pregnancies lead to successful deliveries without significant maternal morbidity [13]. However, numerous patients in previous studies and case reports exhibit diverse factors complicating direct analysis or comparison. These factors encompass advancing maternal age, compromised uterine walls, prior miscarriages, past uterine surgeries, varying interventionalist skills, undisclosed fertility intentions, and other unidentified causes of infertility. The precise fertility rate after undergoing UFE or UAE remains unclear; however, an estimated value of approximately 38.3% is derived from the available publications [12]. However, the studies above can be extrapolated for use in UAEs on a different pathology.

#### 3.5.4. Surgical Management

Though historically, surgical management with hysterectomy has been the treatment of choice for massive hemorrhage secondary to AVMs, definitive treatment with hysterectomy is often not desired. Many women with AVMs tend to be of reproductive age and may not have completed childbearing. Furthermore, hysterectomy is major abdominal surgery that carries with it much morbidity, especially when performed in the postpartum period.

Nonetheless, if embolization as the first‐line treatment were to fail, the recommendation for definitive management of an AVM would be hysterectomy [1, 11].

Finally, there are case reports citing surgical management of AVMs via laparoscopic ligation of internal iliac arteries. However, the role of this form of surgical management has not yet been established [14, 15].

The clinical implications of this algorithm are that it establishes a clear pathway for OBGYNs to navigate patients who are at risk of AVM or if there is a concern on imaging for AVM of the uterus. It also allows for multidisciplinary management with IR to allow for timely intervention if needed.

While our study lays the foundation for clinical management of concern for AVM in setting of postpregnancy bleeding, much is still left to be explored on further characterization of AVM on imaging, diagnostic hysteroscopy, utility of progestin therapy for conservative management, and pregnancy after embolization of AVM. These are all areas of future research that would provide clarity for further management.

Strength of this study is that it is the first to propose an algorithm of this nature for clinical management of postpregnancy bleeding when the differential includes RPOCs versus AVM. It proposes this algorithm in a very adaptable manner that is easy to understand and follow and emphasizes the collaborative nature in managing postpregnancy patients that present with bleeding. A limitation of this study is that it uses cases of patients to extrapolate best practices. A future randomized control trial would be needed to further assess the validity of this algorithm in triaging patients appropriately and preventing the morbidity of hysterectomy.

## 4. Conclusion

Much is left to be explored about the best treatment course for conservative management of AVMs. The role for better characterization via imaging, medical management for AVMs with lower PSV, best techniques for IR‐guided embolization, and even alternative surgical methods besides hysterectomy remain to be further explored. Nonetheless, our paper describes an initial attempt at creating a treatment algorithm for suspected AVMs to allow OBGYNs and IRs a means to guide patient care.

## Consent

This case series was reviewed and approved by the Institutional Review Board; approval was obtained on 11/13/2023, STUDY20231300. The IRB determined that this study met criteria for waiver of informed consent because all patient information was deidentified, and no identifiable protected health information is disclosed. The patients provided general consent for use of their deidentified clinical data for research purposes at the time of admission to our institution.

## Conflicts of Interest

The authors declare no conflicts of interest.

## Funding

No financial support was used for this research.

## Supporting Information

The supporting section includes a table version of the cases highlighted above for ease of reference.

## Supporting information


**Supporting Information** Additional supporting information can be found online in the Supporting Information section.

## Data Availability

The research data are not shared.
