# Supplementary material for: A Practice Algorithm for Distinguishing Uterine Arteriovenous Malformation in Postpregnancy Hemorrhage
Source: Case Rep Med. 2025 Dec 22;2025:2450436. doi: 10.1155/carm/2450436 (PMC12723177; doi:10.1155/carm/2450436)
Supplement: Supplementary file 1 — Supporting Information Additional supporting information can be found online in the Supporting Information section. [file CARM-2025-2450436-s001.docx]

| Case | Clinical Characteristics | Imaging Features | Outcome / Final Diagnosis |
| --- | --- | --- | --- |
| Case 1 | 41-year-old G3P1021  • Prior cesarean and D&C for 10-week missed abortion  • Presented 4 weeks later with heavy persistent bleeding  • Hemoglobin drop 12 → 9 g/dL | **TVUS:** Hypervascular lesion; concern for AVM vs RPOC   \| **MRI:** Multiple serpiginous flow-voids in uterine wall/endometrium/parametrium \| \| --- \| \| **Angiography:** Early venous drainage; serpentine AVM vessels \| | \| • Underwent uterine artery embolization \| \| --- \| \| • Bleeding resolved; discharged stable \| |
| Case 2 | 35-year-old G2P2002   \| • Term SVD with manual placental removal + PPH \| \| --- \| \| • Returned on PP day 4 (light bleeding), PP day 10 (D&C for RPOC; pathology: focal accreta) \| \| • Recurrent heavy bleeding on PP day 27 \| | **TVUS:** Hypervascular lesion  **CT Angiogram:** Enhancing serpiginous uterine artery branches with early venous drainage  **Angiography:** Left-sided AVM confirmed | \|  \| \| • Underwent uterine artery embolization \| \| --- \| \| • Bleeding improved to spotting; discharged \| \|  \|  \| \| --- \| --- \| --- \| --- \| --- \| --- \|  \|  \| \| --- \| |
| Case 3 | 32-year-old G2P2   \| • Vaginal delivery complicated by cervical laceration \|  \|  \|  \| \| --- \| --- \| --- \| --- \|  \| • Tissue adherent to myometrium intra-op; biopsy: RPOC \|  \|  \|  \| \| --- \| --- \| --- \| --- \|  \| • Continued moderate bleeding by PP day 16 \| \| --- \| | **TVUS:** Findings consistent with RPOC; no high-flow AVM features | \| • Prophylactic UAE performed due to accreta concern \|  \|  \|  \| \| --- \| --- \| --- \| --- \|  \| • Bleeding fully resolved \| \| --- \| |
| Case 4 | 23-year-old G3P1021   \| • Persistent bleeding for 1 month after 6-week SAB \|  \|  \|  \| \| --- \| --- \| --- \| --- \|  \| • Hemodynamically stable; Hgb 11.1; β-hCG 165 mIU/mL \| \| --- \| | **TVUS:** Thickened endometrium; focal heterogeneous tissue with turbulent flow at endometrial–myometrial junction | \| • Differential: RPOC vs AVM \|  \|  \|  \| \| --- \| --- \| --- \| --- \|  \| • Treated with misoprostol \|  \|  \|  \| \| --- \| --- \| --- \| --- \|  \| • Follow-up TVUS: thin endometrium; bleeding resolved \| \| --- \| |
| Case 5 | 34-year-old G1P1   \| • Term vaginal delivery with retained placenta requiring manual removal + D&C \|  \|  \|  \| \| --- \| --- \| --- \| --- \|  \| • Presented PP day 24 with heavy vaginal bleeding but stable vitals/Hgb \| \| --- \| | **TVUS:** Thickened endometrium with hypervascular heterogeneous material  **IR review:** Low suspicion for AVM | \| • Hysteroscopic resection of retained tissue \|  \|  \|  \| \| --- \| --- \| --- \| --- \|  \| • Bleeding resolved completely \| \| --- \| |

**Table 1: Review of clinical cases and characteristics**
